# Stem cell enriched lipotransfer reverses the effects of fibrosis in systemic sclerosis

**DOI:** 10.1371/journal.pone.0218068

**Published:** 2019-07-17

**Authors:** Aurora Almadori, Michelle Griffin, Caroline M. Ryan, Debbie F. Hunt, Esther Hansen, Ravi Kumar, David J. Abraham, Christopher P. Denton, Peter E. M. Butler

**Affiliations:** 1 UCL Division of Surgery & Interventional Science, University College London, London, United Kingdom; 2 Department of Plastic Surgery, Royal Free London NHS Foundation Trust Hospital, London, United Kingdom; 3 The Charles Wolfson Center for Reconstructive Surgery, Royal Free London NHS Foundation Trust Hospital, London, United Kingdom; 4 Centre for Rheumatology, UCL Division of Medicine and Royal Free London NHS Foundation Trust Hospital, London, United Kingdom; Temple University, UNITED STATES

## Abstract

Oro-facial fibrosis in systemic sclerosis (Scleroderma;SSc) has a major impact on mouth function, facial appearance, and patient quality of life. Lipotransfer is a method of reconstruction that can be used in the treatment of oro-facial fibrosis. The effect of this treatment not only restores oro-facial volume but has also been found to reverse the effects of oro-facial fibrosis. Adipose derived stem cells (ADSCs) within the engrafted adipose tissue have been shown to be anti-fibrotic in SSc and are proposed as the mechanism of the anti-fibrotic effect of lipotransfer. A cohort of 62 SSc patients with oro-facial fibrosis were assessed before and after stem cell enriched lipotransfer treatment. Clinical evaluation included assessment of mouth function using a validated assessment tool (Mouth Handicap in Systemic Sclerosis Scale-MHISS), validated psychological measurements and pre and post-operative volumetric assessment. In addition, to understand the mechanism by which the anti-fibrotic effect of ADSCs occur, SSc derived fibroblasts and ADSCs from this cohort of patients were co-cultured in direct and indirect culture systems and compared to monoculture controls. Cell viability, DNA content, protein secretion of known fibrotic mediators including growth factor- β1 (TGF β-1) and connective tissue growth factor (CTGF) using ELISA analysis and fibrosis gene expression using a fibrosis pathway specific qPCR array were evaluated. Mouth function (MHISS) was significantly improved (6.85±5.07) (p<0.0001) after treatment. All psychological measures were significantly improved: DAS 24 (12.1±9.5) (p<0.0001); HADS-anxiety (2.8±3.2) (p<0.0001), HADS-depression (2.0±3.1) (p<0.0001); BFNE (2.9 ± 4.3) (p<0.0001); VAS (3.56±4.1) (p<0.0001). Multiple treatments further improved mouth function (p<0.05), DAS (p<0.0001) and VAS (p = 0.01) scores. SSc fibroblast viability and proliferation was significantly reduced in co-culture compared to monoculture via a paracrine effect over 14 days (p < 0.0001). Protein secretion of transforming growth factor (TGF-β1) and connective tissue growth factor (CTGF) was significantly reduced in co-culture compared to monoculture (p < 0.0001). Multiple fibrosis associated genes were down regulated in SSc co-culture compared to monoculture after 14 days including Matrix metalloproteinase-8 (MMMP-8), Platelet derived growth factor-β (PDGF-β) and Integrin Subunit Beta 6 (ITG-β6). Autologous stem cell enriched lipotransfer significantly improved the effects of oro-facial fibrosis in SSc in this open cohort study. Lipotransfer may reduce dermal fibrosis through the suppression of fibroblast proliferation and key regulators of fibrogenesis including TG-β1 and CTGF. Our findings warrant further investigation in a randomised controlled trial.

## 1. Introduction

Systemic Sclerosis (Scleroderma;SSc) is a complex multisystem disease characterised by autoimmunity, microvascular dysfunction and fibrosis of the skin and internal organs [1–2]. It has a female predominance and the usual age of onset is between 30 and 60 years [1]. Almost all patients with SSc have skin fibrosis despite the disease heterogeneity. Skin fibrosis is the clinical hallmark of SSc, particularly in the face [3–5]. Although SSc has a high mortality rate due to internal organ complications [6], there is also a very high disease burden due to its impact on facial appearance and oro-facial function.

The main oro-facial features of SSc include skin induration, thickening and atrophy, retraction of the lips, microstomia, perioral fissuring, telangiectasia, and atrophy of the nasal alae [7,8]. In more severe cases, microstomia inhibits lip and mouth closure, which leads to breathing and chewing impairment [9,10], and impacts oral hygiene and dental treatment [8]. Fibrosis of the salivary and lacrimal glands can also lead to xerostomia and xerophthalmia [8]. Appearance and functional changes of SSc has a significant negative impact on patients’ quality of life, leading to social disability, isolation and psychological distress [11,12]. Facial changes were ranked as the most worrying aspect of the condition and was considered more important than any internal organ involvement by the majority of patients affected [13].

Current avaIlable therapies for SSc focus on life-threatening complications arising from organ involvement. An effective disease modifying therapy is lacking and no treatment is available to reverse skin fibrosis [14]. Autologous lipotransfer is considered a standard reconstructive surgical technique for reconstruction of contour deformities [15,16], and is minimally invasive with low morbidity. Our group and others have used autologous lipotransfer as a successful treatment option in different fibrotic conditions such as SSc, hypertrophic scars, burns, radiation-induced fibrosis, lichen sclerosus, and Dupuytren’s disease [17–20]

Dermal fibrosis is a complex pathological process caused by the deposition and accumulation of extracelular matrix, mainly type I collagen in the dermis [21]. Fibroblasts are the key contributors to fibrosis in patients with SSc. Dermal fibroblasts isolated from SSc patients have shown to exhibit increased prolifeartion, synthesis of collagen and decreased collagenase activity [21–23]. The upregulation of collagen by SSc fibroblasts involves the alteration of several molecular regulators including cytokines and transcription factors. To date, transforming growth factor-beta-1 (TGF-β1) and connective tissue growth factor (CTGF) have been shown to play a significant role in the pathway of dermal fibrosis [21]. TGF-β1 is a multifunctional cytokine that regulates growth and differentiation of several cell types. TGF-β1 binds to specific proteins (receptors) on the cell membrane, which signal the association of Smad signalling proteins to activate collagen synthesis [21]. The Smad proteins are thought to be one of the most potent mediators of upregulated collagen activity in SSc fibroblasts [21]. Connective tissue growth factor (CTGF) is a 36–38 kDa peptide, partially controlled by TGF-β and itself regulates multiple celular processes including mitrogenesis, chemotaxis, extracelular matrix (ECM) production, angiogenesis and apoptosis [21]. In vitro, CTGF enhances fibroblast activation and ECM production as a downstream mediator of TGF-β1 [21].

The effector mechanism that is responsible for the improvement in fibrosis following stem cell enriched lipotransfer is not known. Adipose-derived stem cells (ADSCs) are a multipotent population of progenitor cells that are found within the adipose tissue [24]. They have been identified as the potential effector cell in stem cell enriched lipotransfer. In addition to their multipotency, this population exhibits paracrine proangiogenic, anti-inflammatory and immunomodulatory activities [25,26]. To understand the mechanism by which ADSCs may reduce fibrosis in the scleroderma patients, we co-cultured ADSCs with scleroderma fibroblasts in culture and evaluated their proliferation, effect on gene and protein expression of known fibrotic growth factors and cytokine mediators.

The aim of this study was to analyse the effect of autologous adipose stem cell-enriched lipotransfer on the effects of oro-facial fibrosis in a large cohort of SSc patients. As a primary outcome we aimed to assess the effect of autologous lipotransfer on mouth function. Secondary outcomes aimed to assess the psychological status, volumetric facial changes and clinical outcome. As a secondary outcome we aimed to better understand the mechanism by which ADSCs may have reduced fibrosis in the scleroderma patients co-culturing ADSCs with scleroderma fibroblasts and evaluating their proliferation, effect on gene and protein expression of known fibrotic growth factors and cytokine mediators. Lastly preliminary in vitro data demonstrated that in tissue culture ADSC modulated fibrobast properties, including attenuation of some of the key profibrotic characteristics of SSc fibroblasts in vitro. This provides a potential mechanism for the clinical benefit observed after autologous stem cell enriched lipotransfer in scleroderma.

## 2. Materials and methods

### 2.1 Clinical analysis

#### 2.1.1 Participants

62 patients with systemic sclerosis (SSc) were included in this series (Table 1). Patients included in the study fulfilled the following inclusion criteria, (1) patients with a confirmed diagnosis of either diffuse or limited cutaneous systemic sclerosis, (2) adult patients (18–65 years of age), (3) stable SSc disease for at least 2 years, (4) stable lung and cardiac function as shown by echocardiogram and lung function tests, (5) to be able to safely undergo a general anaesthesia and (6) documented oro-facial dysfunction as assessed by MHISS.

**Table 1:** Patient demographics.

Number of patients	62
Age mean (± SD)	56 (±11.59)
Sex	61 Female, 1 Male
Duration of SSc mean (±SD)	15 years (±8.81)
Subset (number of patients)	dcSSc (26), lcSSc (36)
Concurrent immunosuppression (number of patients)	Yes (31), No (31)
**Major Drug treatment**	
Mycophenolate Mofetil (MMF)	14
Methotrexate (MTX)	6
Other	11

*SSc* Systemic Sclerosis, *dcSSC* diffuse cutaneous Systemic Sclerosis, *lcSSc* limited cutaneous Systemic Sclerosis.

All patients included presented with oro-facial changes associated with SSc such as hardened and taut facial skin, loss of tissue volume, retracted and tightened lips and microstomia. This study was performed with ethical approval and informed consent was obtained from all patients participating in the study. This study was approved by the Hampshire B Research Ethics Committee (REC reference: 16/SC/0669, IRAS project ID: 196386). Informed written consent was obtained from all patients participating in the study in accordance with the research ethics committee approval.

#### 2.1.2 Surgical intervention

The standardized autologous lipotransfer surgical intervention was performed as described by our group and others [17,18]. The lipoaspirate was obtained from abdominal area or from thighs according to that described by Syndey Coleman et al [15–16]. Using a 15 cm x 3 mm disposable cannula connected to a 10cc Luer Lock syringe the adipose was harvested from the superficial layers of the subcutaneous fat of the abdomen. Adequate fat graft volume was obtained from the participants despite their fibrosis. After harvesting, the lipoaspirate was enriched with stem cells through centrifugation. Our group and others have demonstrated that centrifugation at 3000rpm for 3 mins of the adipose tissue enriches fat with ADSC’s in the distal portion of the lipoaspirate [27–29]. The proximal lipoaspirate graft was discarded along with the free oil and blood segments and only the distal lipoaspirate graft was used for injections. The stem cell enriched adipose tissue was transferred into a 1ml Luer-Lock syringes connected to 9 cm by 2mm blunt disposable cannulae. The stem cell enriched fat was directly injected into the fibrotic oro-facial tissues using a minimally invasive technique as described by Coleman et al [15] using small skin incisions (2mm). The lipoaspirate was injected slowly using multiple passages with injection of lipoaspirate on withdrawal of the cannula. The volume of graft injected into each facial area was recorded.

#### 2.1.3 Assessment of mouth function

Mouth function was assessed pre and post-operatively using the *Mouth Handicap in Systemic Sclerosis Scale* (MHISS) [30]. MHISS is a validated scale assessing the handicap associated with mouth disability in SSc. It consists of 12 items each scored from 0 to 4, with a total score ranging from 0 (minimal handicap) to 48 (maximal handicap). The 12 items are grouped in three subscales: part 1 examines the handicap related to reduced mouth opening and dental issues; part 2 assesses the handicap related to mouth dryness; part 3 is related to aesthetic concerns [30,31].

#### 2.1.4 Assessment of psychological status

Psychological status was assessed pre and post-operatively using validated questionnaires. The *Derriford Appearance scale* (DAS24) examines the frequency of avoidant or maladaptive behaviours and distress related to an appearance concern(s), with strong psychometric properties with social anxiety, shame and negative affect [32]. Higher scores suggest higher levels of distress and social avoidance. The *Hospital Anxiety and Depression Scale* (HADS) is a validated 14 item self-report scale measuring current affective psychological functioning, standardized on both general and hospital populations and widely used in research with patients who have physical health problems [33], including in our previous study with patients with facial disfigurement [34]. It consists of two subscales, one measuring levels of anxiety and the other depression. The *Brief Fear of Negative Evaluation Scale* (BFNES) is a validated 12 items self-report scale examining the extent to which a person may be pre-occupied by other people’s opinions regarding themselves [35]. Eight of the items are positively scored and four are negatively scored in order to reduce the risk of response bias [36], and potential scores range from 12 to 60, with high scores indicating greater fear of negative evaluation. We also used three visual analog scales (VAS) used for subjective ratings of mood, emotion, distress on which the patient ranks the perceived noticeability of their disfigurement, including to an observer, and their pre-occupation with this appearance concern. Higher rankings suggest high levels of noticeability [34, 37].

#### 2.1.5 Assessment of volume augmentation

Pre- and post-operative 3D scans were recorded using the 3dMD system to measure pre- and post-operative volumetric changes. The 3dMD Vultus software was used to calculate facial volumetric changes. The pre- and post-operative 3D scans were superimposed and aligned by rotation using the XYZ rotational coordinates. The alignment precision was calculated by the root mean square (RMS) error, which shows the variation between the two surfaces. An RMS value of 0.5 mm or less was considered acceptable to obtain accurate alignments (www.3dmd.com). Volume change was calculated in nose, upper lip, lower lip, nasolabial folds, cheeks, and chin. Each area was measured three times and the average of the three measurements was recorded. A colour map was then generated to represent the relative volume change between the pre- and post-operative image. Volume change was then compared to volume injected and percentage of volume retained over time was calculated.

#### 2.1.6 Photograph assessment

Pre- and post-operative 2D photographs of each patient were evaluated by three independent blinded clinical observers. Each image was graded according to appearance, representing oro-facial disease severity as follows: severe, severe-moderate, moderate, moderate-mild and mild.

### 2.2 In vitro analysis

#### 2.2.1 Isolation and Culture of Adipose Derived Stem Cells (ADSCs)

Three female patients who were being followed up for clinical analysis were also included in the *in vitro* analysis. All participants gave informed written consent. This study was approved by the North Scotland ethical review board, reference number 10/S0802/20. Following the adipose harvesting in three participants (age range, 45–55 years) as described above, lipoaspirate from the abdomen was used to isolate ADSCs for in vitro analysis. The three donors had an average duration of disease of 10 years and all of these patients had improvement in oro-facial fibrosis following lipotransfer. ADSCs were isolated from the lipoaspirate samples according to a modified method as previously described [28]. In brief, after the removal of fibrous tissue and visible bloods vessels, lipoapirate samples were cut into small pieces and digested in Dulbecco's Modified Eagle's Medium/Nutrient Mixture F-12 Ham (DMEM/F12) containing 300 U/ml crude collagenase II (Invitrogen, Life Technologies Ltd, Paisley, UK) for 30 min in an incubator (37°C, 5% CO_2_). Following this the digest was filtered though cell strainers and then centrifuged. After red cell lysis the ADSC pellet was then resuspended and expanded into cell culture. Cells were maintained in culture DMEM/F12 supplemented with 10% Foetal Bovine Serum (FBS) and 1% antibiotic solutions for 3 passages at 37°C in a humidified atmosphere of 5% CO_2_ before being using for analysis.

#### 2.2.2 Culture of Human Dermal Fibroblasts (HDFs)

Scleroderma fibroblast (SSc HDF) were grown by explant culture from 4-mm^3^ punch biopsies of three female donors (age range, 45–60 years) with diffuse scleroderma. All three donors had duration of SSc disease for 10 years (range, 9–12 years). Biopsies were taken for clinical or research purposes with full informed consent, and this study was approved by the London-Hampstead National Research Ethics Committee (HRA reference 6398). Normal control human dermal fibroblasts (HDF) were obtained from the European Collection of Cell culture (ECACC). Both SSc-HDF and HDF were maintained in Dulbecco's Modified Eagle's Medium/Nutrient Mixture F-12 Ham (DMEM/F12) with 10% Foetal Bovine Serum (FBS) and 1% antibiotic solutions (Sigma, UK). In this study, SSc derived fibroblasts were referred to as SSc and control derived fibroblasts were referred to as HDF.

#### 2.2.3 Co-culture set up

For all analysis, three different co-culture experiments were performed to evaluate the effect of ADSCs on scleroderma and normal fibroblast behaviour. Firstly, 2.5 x 10^4^ ASDCs were co-cultured with 2.5 x 10^4^ HDFs or SSc-HDF in a 6 well plate for 14 days and referred to as *direct culture*. Secondly, using a transwell insert 2.5 x 10^4^ ASDCs in the top chamber were co-cultured with 2.5 x 10^4^ HDFs or 2.5 x 10^4^ SSc-HDF in the bottom chamber for 14 days and referred to as *indirect culture*. The transwell insert had pore of 0.4 μm diameter to permit movement of cytokines between chambers but prevent cell migration. Thirdly, ADSCs were grown for 48 hours in DMEM/F12 supplemented with 10% Foetal Bovine Serum (FBS) and 1% antibiotic solutions for 48 hours. After 48 hour the medium was harvested and cellular debris removed by centrifugation at 3000 g for 10 minutes. Medium was either used immediately or stored frozen at -70°C for later experiments and referred to as *conditioned medium* (CM). As control SSc-HDF, HDF and ADSC monocultures were also set up for direct, indirect and conditioned medium experiments.

#### 2.2.4 Cell viability and DNA content

Cell viability and proliferation were assessed by Alamar Blue and DNA assay respectively as described previously [28]. In brief, the commercial available assay Alamar Blue^TM (^Life Technologies, UK) was used as per manufacturer instructions to assess cell viability. After 4 hours of incubation with 10% alamar blue dye, 100 μl of media was place into 96 well plates and fluorescence was measured at excitation and emission wavelength of 530 and 620 nm using Fluoroskan Ascent FL, (Thermo Labsystems, UK) (n = 6). To assess proliferation, Fluorescence Hoechst DNA Quantification Kit was utilized to quantify the DNA content (Sigma, UK). The assay was used according to manufacturer instructions and the fluorescence was measured at 360 nm and emission at 460 nm using Fluoroskan Ascent FL, (Thermo Labsystems, UK) (n = 6). Each experiment was performed in triplicate. For the direct and indirect assays both viability of both cell populations was assessed.

#### 2.2.5 Enzyme Linked Immunosorbant Assay (ELISA)

Eliza assay were used to investigate the paracrine effect of co-culturing SSc fibroblasts with ADSCs on the effect of cytokine TGF-β1 and CTGF. The Quantikine ELISA kit targeting TGF-β1 (R&D systems, UK) and ELISA Development kit targeting CTGF (Pepro Tech, UK) was performed according to manufacturing instructions. In brief, three co-culture experiments were set up as described earlier. At day 2, 4, 7, 10 and 14 medium were removed and used for analysis (n = 6).

#### 2.2.6 Quantitative Real-Time Polymerase Chain Reaction (qPCR)

The effect of co-culturing SSc fibroblasts with ADSCs on fibrotic gene expression was studied using a RT2 Human Fibrosis PCR Array (SabioSciences, Qiagen), a system that simultaneously profiles expression of 84 fibrosis specific genes. After 14 days the gene expression of SSc monoculture and direct SSc and ADSC co-culture was compared (n = 3). Each experiment was performed in triplicate. Data presented is the fold change normalized to the 5 hours keeping genes. Cell lysis and RNA purification was performed using the RNeasy mini kit (Qiagen). The cDNA synthesis was performed using the RT2 First Strand Kit (Qiagen). RT2 Profiler PCR Arrays in a 100-well Rotor Disc format were obtained from Sabiosciences, Qiagen. Each disc contains primers targeting a total of 5 housekeeping genes, 84 fibrosis-associated genes, 3 positive controls, 3 negative controls, and 3 wells for balancing. The RT2 ROX FAST Mastermix (Qiagen), containing *Taq* Polymerase, was used to prepare samples prior to commencing qPCR. A Corbett RotorGene-6000 (Qiagen) was used for real-time sample analysis. The threshold cycle (CT) for each well was obtained using real-time cycler software. A log view of amplification plots was generated and a threshold value was selected in the linear phase of the plot. The relative fold-change in fibrosis associated gene expression compared to housekeeping genes for ADSC-SSc co-culture and SSc monoculture was calculated using the ΔΔCT method. The difference in fold change of ADSC-SSc co-culture and SSc monoculture was then calculated (n = 3).

#### 2.2.7 Statistical analysis

Inter comparisons between pre- and post-treatment were analysed statistically using paired t-test with nonparametric Wilcoxon test (Prism6 Software). All other comparisons were analysed using non-paired t-tests. Tests were two-tailed with a confidence interval of 95%. The average and standard deviation (SD) was calculated.

## 3. Results

### 3. 1. Clinical analysis

#### 3.1.1 Participants

Of the 62 patients, mean age was 56 (±11.59) and 98% were female. The mean follow up after the last treatment was 12.41 (±8.64) months (median 8 months, range 6–53 months). 29 patients received ≤2 treatments and 33 patients received ≥3 treatments. Patients received on average 3 lipotransfer procedures (median 2 treatments, range 1–10 patients) (Table 1).

Of the 62 patients, 31 patients were on immunosuppressant medication and 31 patients were not. The immunosuppressant medication was apart of the participant’s standard routine care, determined by clinical guidelines. 58% were affected by limited cutaneous systemic sclerosis (lcSSc) and 42% by diffuse cutaneous systemic sclerosis (dcSSc).

#### 3.1.2 Tolerability and adverse events

The autologous lipotransfer procedure was overall well tolerated. Normal post-operative sequelae occurred (bruising, swelling and tenderness of donor site). These resolved within 14 days. We experienced only one case of superficial wound infection occurred at the recipient site, which responded to oral antibiotic therapy and no further surgical intervention was required. No other complication was observed.

#### 3.1.3 Mouth function outcomes

Patients reported a significant improvement in mouth function following treatment (6.85 ± 5.07) (p < 0.0001) (Table 2). Analysis of the three sub-domains of the MHISS showed that there was a significant improvement in each of the three sub domains. The mouth opening domain (3.4 ± 2.64) contributed to 50% of the overall MHISS score. The aesthetic concerns domain contributed 28.5% (1.95 ± 1.44) and the sicca syndrome domain 21.6% (1.5 ± 1.21) (Table 2).

**Table 2:** Effect of lipotransfer treatment on mouth function measured by the Mouth Handicap in Systemic Sclerosis scale (MHISS).

MHISS	Pre-op score	Post-op score	Change in score	p value
Overall (n = 62)	31.27 ± 6.08	24.4 ± 7.11	6.85 ± 5.07	0.0001
Mouth opening subset (n = 62)	15.9 ± 3.08	12.5 ± 3.6	3.4 ± 2.64	0.0001
Sicca syndrome subset (n = 62)	7.0 ± 1.39	5.5 ± 1.58	1.5 ± 1.21	0.0001
Aesthetic concerns subset (n = 62)	8.42 ± 1.7	6.46 ± 1.99	1.95 ± 1.44	0.0001
≤2 LT procedures (n = 29)	29.82 ± 6.9	24.41 ± 7.04	5.41 ± 4.62	0.0001
≥3 LT procedures (n = 33)	32.54 ± 5.0	24.42 ± 7.28	8.12 ± 5.17	0.0001
Change in score after ≤2 LT compared to ≥ 3 LT	-	-	-	0.0368

Data are presented as mean ±SD, n = number of patients, p≤0.05 was considered significant. *LT* Lipotransfer.

Results were subdivided and compared regarding: the number of treatment the patients received (≤2 versus ≥3 treatments); concomitant immunosuppressant medication (patients on immunosuppressant medication versus patients that were not); the disease subset (lcSS versus dcSS). Patients that received ≥3 treatments (33 patients) had a higher improvement in mouth function (8.12 ± 5.17) compared to patients that received ≤2 treatments (29 patients) (5.41 ± 4.62) (p = 0.03) (Table 2). When we compared patients that were on immunosuppressant medication (31 patients) to patients that were not (31 patients), we found no difference in MHISS scores (p = 0.18). When we compared patients with lcSSc (36 patients) to patients with dcSSc subset (26 patients) we found no difference in MHISS scores (p = 0.90).

#### 3.1.4 Psychological outcomes

Patients reported a significant improvement in all the psychological measures following treatment: VAS for noticeability of disfigurement (3.56 ± 4.1) (p<0.0001); DAS24 scores (12.1 ± 9.5) (p<0.0001) that measures the level of psychological distress related to physical appearance; HADS-A score (2.8 ± 3.2) (p<0.0001) that measures levels of anxiety, HADS-D score (2.0 ± 3.1) (p<0.0001) that measures levels of depression; BFNE scale (2.9 ± 4.3) (p<0.0001) that measures the perceived negative judgment from others (Table 3).

**Table 3:** Effect of lipotransfer treatment on psychological outcomes.

	Pre-op score	Post-op score	Change in score	p value
VAS (n = 62)	24.16 ± 5.81	20.59 ± 6.37	3.56 ± 4.16	<0.0001
DAS24 (n = 62)	55.91 ± 16.21	43.83 ± 15.41	12.08 ± 9.49	<0.0001
BFNES (n = 62)	39.35 ± 9.34	36.45 ± 9.81	2.91 ± 4.34	<0.0001
HADS-A (n = 62)	10.38 ± 4.32	7.54 ± 3.89	2.83 ± 3.29	<0.0001
HADS-D (n = 62)	8.38 ± 4.53	6.35 ± 3.74	2.03 ± 3.19	<0.0001

Data are presented as mean ±SD, n = number of patients, p≤0.05 was considered significant. Psychological outcomes were evaluated by self-report questionnaires. *VAS* Visual Analog Scale, *DAS24* Derriford Appearance Scale, *BFNES* Brief Fear of Negative Evaluation Scale, *HADS-A* Hospital Anxiety and Depression Scale-Anxiety, *HADS-D* Hospital Anxiety and Depression Scale-Depression.

Results were subdivided and compared regarding: the number of treatment they received; the concomitant immunosuppressant medication; the disease subset (lcSS versus dcSS). Patients that received ≥3 treatments (33 patients) reported a significant improvement (4.6 ± 4.7) in the VAS for noticeability of disfigurement compared to patients that received ≤2 treatments (29 patients) (2.4 ± 3.0) (p = 0.01) (S1 Table). Patients that received ≥3 treatments reported a significant improvement (16.2 ± 9.7) in DAS24 score compared to patients that received ≤2 treatments (7.4 ± 6.9) (p<0.0001) (S1 Table). Patients that received ≥3 treatments (33 patients) sustained their improvement in BFNE (p = 0.38), HADS-A (p = 0.43) and HADS-D (p = 0.48) scores when compared to patients that received ≤2 treatments (29 patients) (S1 Table).

When we compared patients that were on immunosuppressant medication (31 patients) to patients that were not (31 patients), we found no change in VAS (p = 0.63), DAS24 (p = 0.29), HADS-A (p = 0.26), HADS-D ((p = 0.75) or BFNES (p = 0.45) scores. When we compared patients affected by lcSSc (36 patients) to patients affected by dcSSc (26 patients), we found no change in VAS (p = 0.77), DAS24 (p = 0.76), HADS-A (p = 0.41), HADS-D ((p = 0.33) or BFNE (p = 0.83) scores.

#### 3.1.5 Volumetric augmentation outcome

**Fig 1:**
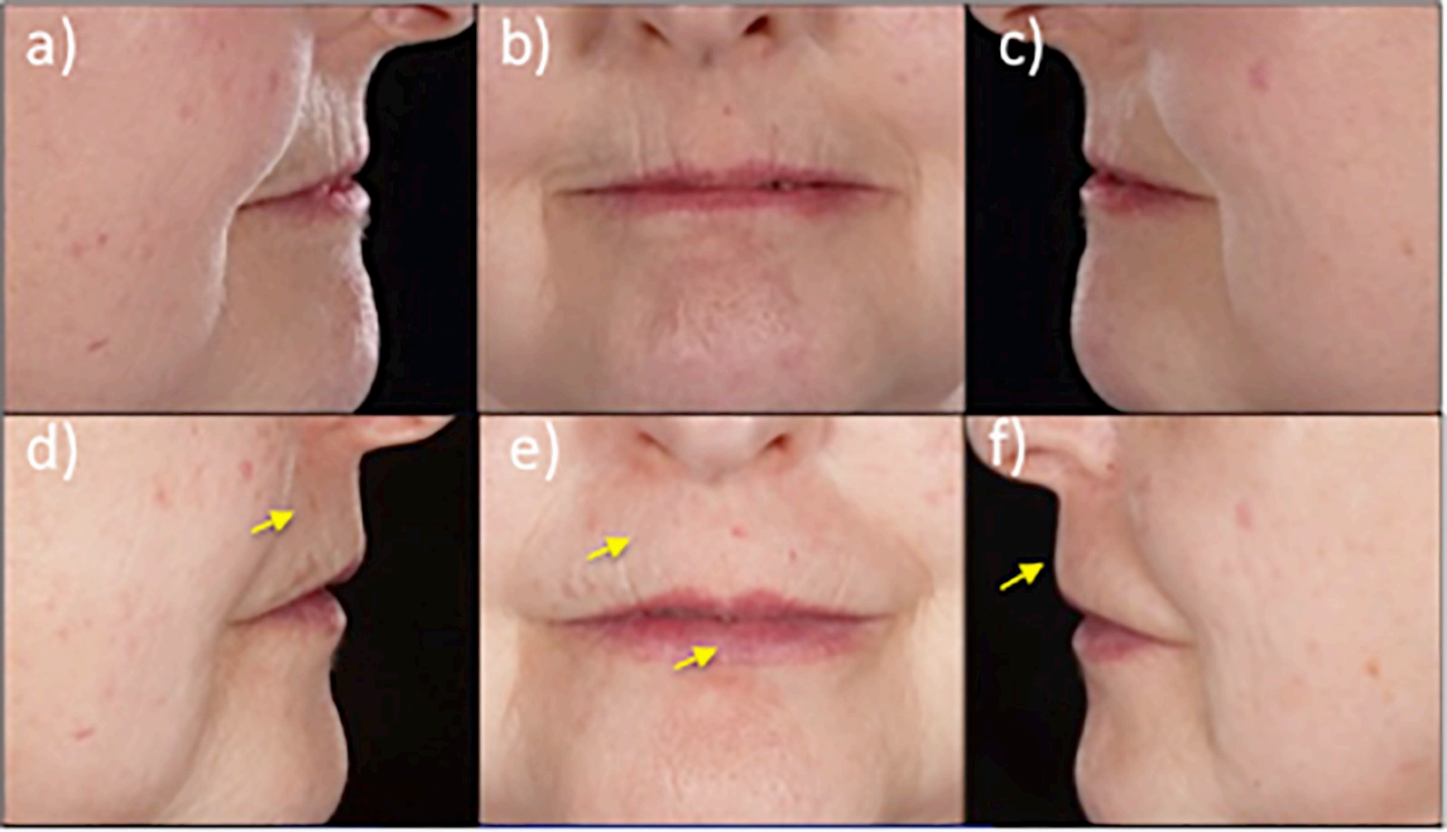
Aesthetic effect of lipotransfer on the peri-oral area. Upper panel: Representative pre-operative images of the peri-oral area of a patient with SSc. Lower Panel: Post-operative images of the peri-oral area of the patient following autologous lipotransfer. The arrows indicate the areas of improvement in vertical furrows and soft tissue bulk.

Fig 1 shows an example of the aesthetic changes in the peri-oral area that were observed after surgical treatment. We found reduction in perioral wrinkling and ridges as well as improvement in lip volumes and increased vermillion show with return to normal lip volume ratios associated with perioral tissue softening (Fig 1).

**Fig 2:**
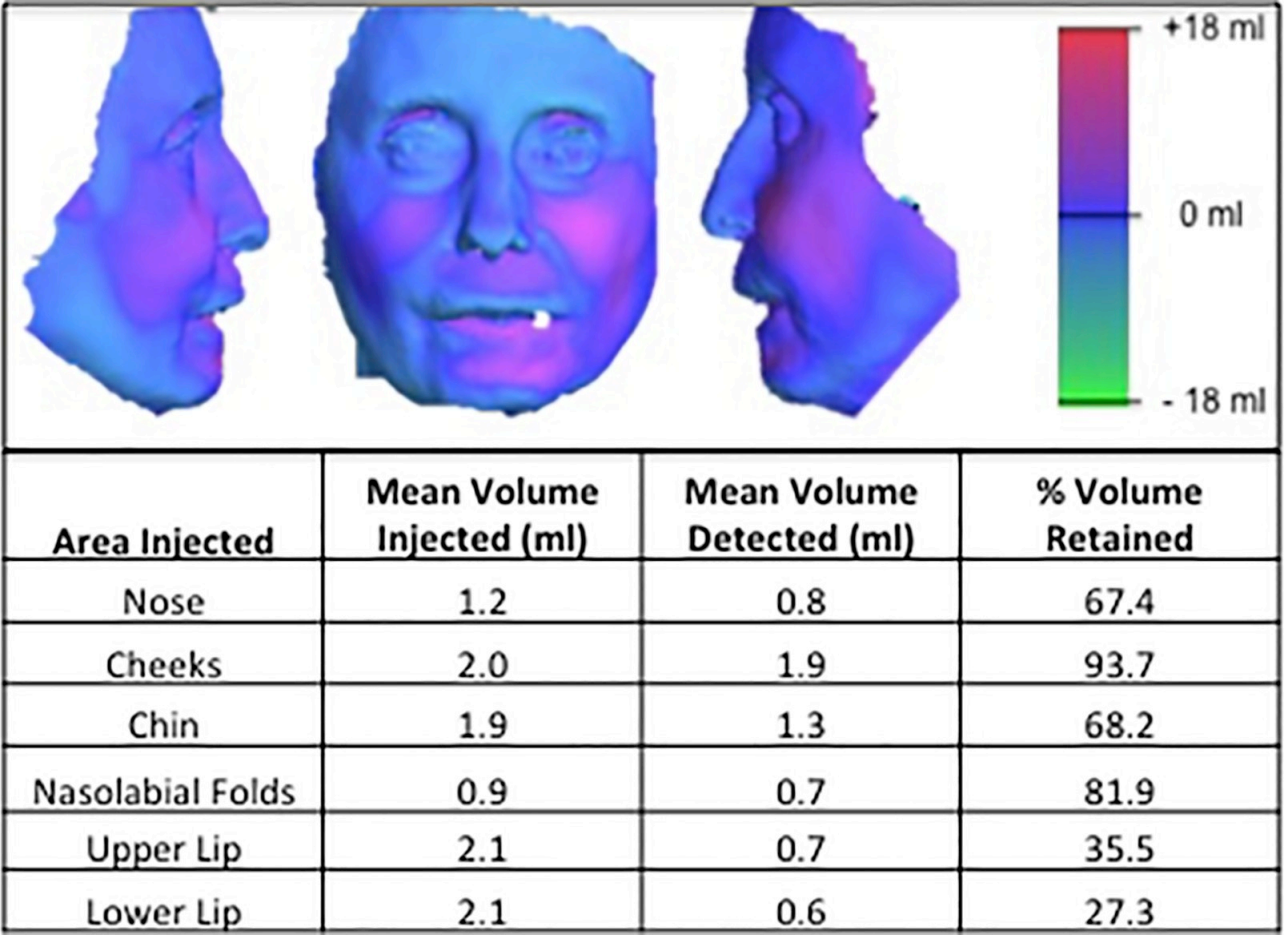
Effect of autologous stem cell enriched lipotransfer on facial volume. Representative heat map generated with 3dMD system demonstrates a change in facial volume after surgical treatment with autologous stem cell enriched lipoaspirate. Volumetric analysis of 3D images was performed with 3dMD system. The volume retained in each facial subunit after autologous stem cell-enriched lipotransfer was calculated as a percentage of the original volume injected.

Increased volume was also observed in the cheeks and nasal area with improved facial contouring. The 3dMD system was used to calculate the change in facial volume of each patient at follow-up. A heat map generated by superimposition of pre-op and post-op images illustrates the change in facial volumes (Fig 2). The cheeks and nasolabial folds retained the greatest percentage of the injected volume, 93.7% and 81.9% respectively. The nose retained 67.4% while the chin retained 68.2%. The upper and lower lips retained the least volume, 35.5% and 27.3% respectively (Fig 2).

#### 3.1.6 Photographic 2-D photograph assessment

Images were graded according to disease severity as severe, severe-moderate, moderate, moderate-mild and mild. Pre-treatment 26% of patients were graded as severe and severe/moderate, 57% were graded as moderate. Post-treatment 0% of patients were graded as severe, 13% were graded severe/moderate and moderate and 40% of patients were graded mild (S2 Table).

### 3.2. In vitro analysis

#### 3.2.1. Cell viability and DNA content

**Fig 3:**
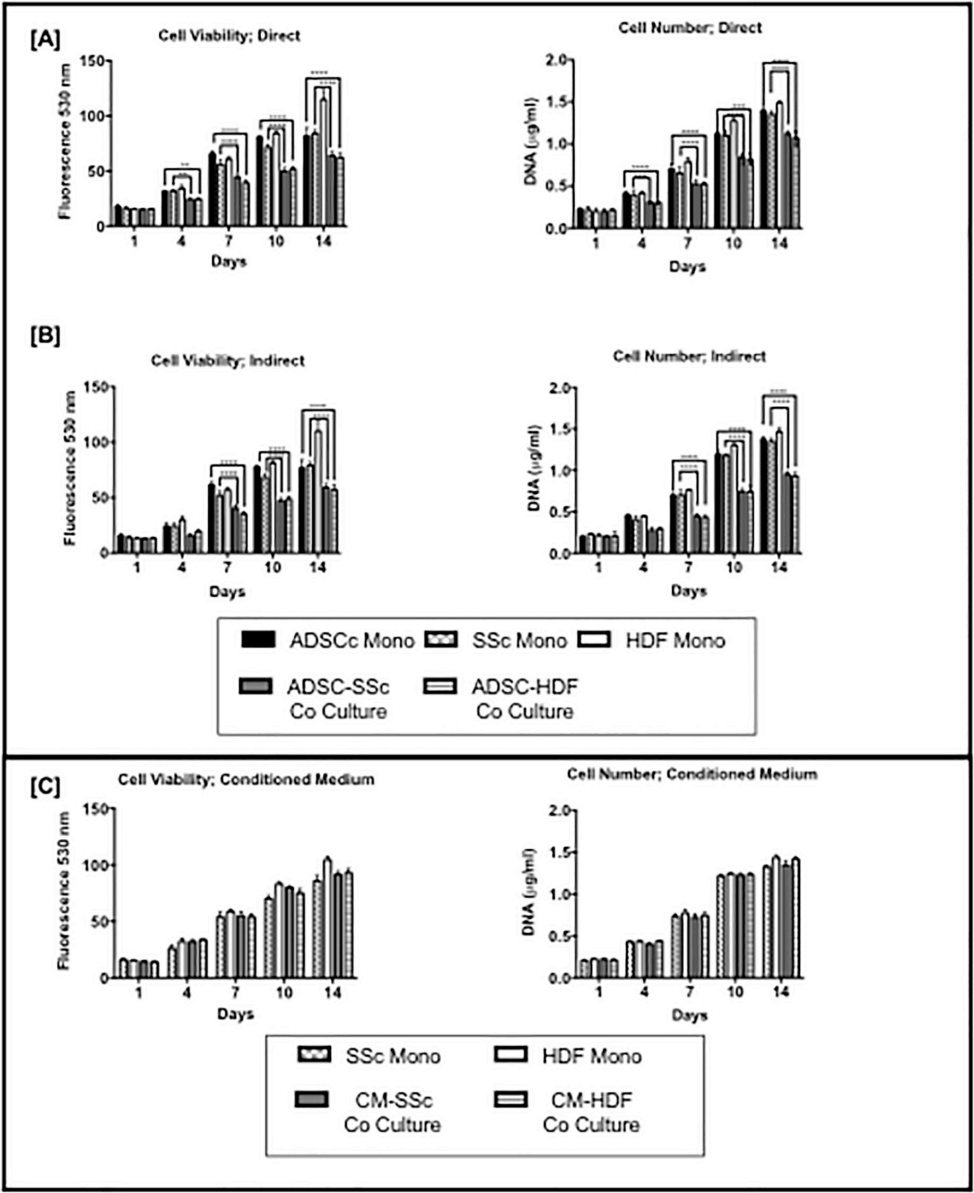
**Effect of monoculture and co-culture on cell viability and DNA content after [A] direct and [B] indirect culture and using [C] conditioned medium.** SSc HDF cell viability and DNA content was significantly lower in co-culture compared to mono-culture on days 4, 7, 10 and 14 in direct and indirect culture (p < 0.001). Conditioned medium had no effect on cell viability and DNA content over 14 days. ADSC Mono; Adipose Derived Stem Cell Monoculture. SSc Mono: Scleroderma Fibroblast Monoculture. HDF Mono; Human Dermal Fibroblast Monoculture. ADSC-SSc Co-Culture. Adipose Derived Stem Cell- Scleroderma Fibroblast Cell Co-culture. HDF Monoculture- Human Dermal Fibroblast Monoculture. * P < 0.05 ** P < 0.01 ***P < 0.001 **** P < 0.0001.

From day 4 to day 14 the cell viability and DNA content of SSc monoculture was significantly higher than ADSC-SSc co-culture (p <0.001) with direct and indirect culture (Fig 3). Similarly, from day 4 to day 14 the cell viability and DNA content of ADSC monoculture was significantly higher than ADSC-HDF co-culture (p <0.001) with direct and indirect culture (Fig 3). The HDF culture demonstrated the highest viability and DNA content over the 14 days (Fig 3). The viability and DNA content of the ADSC and SSc in monoculture was similar over the 14 days (Fig 3). In conditioned medium culture experiments there was no significant differences in the viability and DNA content of SSc and ADSC in co-culture and monoculture over 14 days (Fig 3).

#### 3.2.1. ELISA analysis

**Fig 4:**
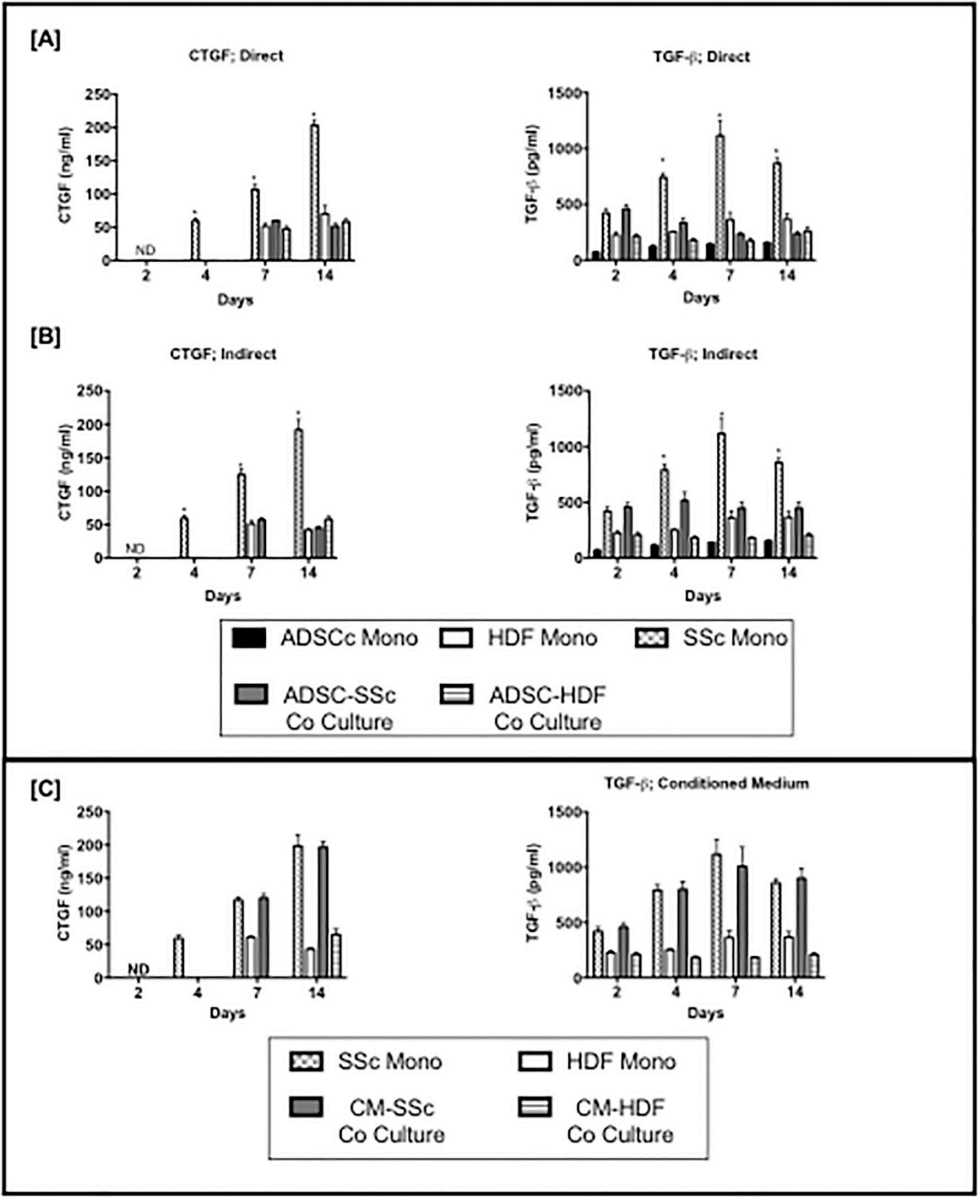
**Effect of monoculture and co-culture on the protein secretion of Transforming Growth Factor-β1 (TGF-β1) and Connective Tissue Growth Factor (CTGF) in [A] direct [B] indirect and using [C] Conditioned Medium.** The protein secretion of TGF-β1 and CTGF was significantly lower in co-culture compared to mono-culture on days 4, 7, 10 and 14 (p < 0.001) in direct and indirect culture using ELISA analysis. Conditioned medium had no effect on protein secretion of TGF-β1 or CTGF over 14 days. ADSC Mono; Adipose Derived Stem Cell Monoculture. SSc Mono: Scleroderma Fibroblast Monoculture. HDF Mono; Human Dermal Fibroblast Monoculture. ADSC-SSc Co-Culture. Adipose Derived Stem Cell- Scleroderma Fibroblast Cell Co-culture. HDF Monoculture- Human Dermal Fibroblast Monoculture. * P < 0.05 ** P < 0.01 ***P < 0.001 **** P < 0.0001.

On day 4, 7 and 14 the protein secretion of TGF-β1 from SSc monoculture was significantly higher than ADSC-SSc co-culture, HDF monoculture, ADSC monoculture and ADSC-HDF co-culture (p <0.001) with direct and indirect culture (Fig 4). On day 4, 7 and 14 the protein secretion of CTGF from SSc monoculture was significantly higher than ADSC-SSc co-culture, HDF monoculture, ADSC monoculture and ADSC-HDF co-culture (p <0.001) with direct and indirect culture (Fig 4). In conditioned medium culture experiments there was no significant differences in the protein secretion of TGF-β1 or CTGF of SSc monoculture and SSc in CM (Fig 4). The protein secretion of TGF-β1 was lower in HDF grown in CM than monoculture at day 7 and 14 (p < 0.001) (Fig 4).

#### 3.2.3 qPCR analysis

**Fig 5:**
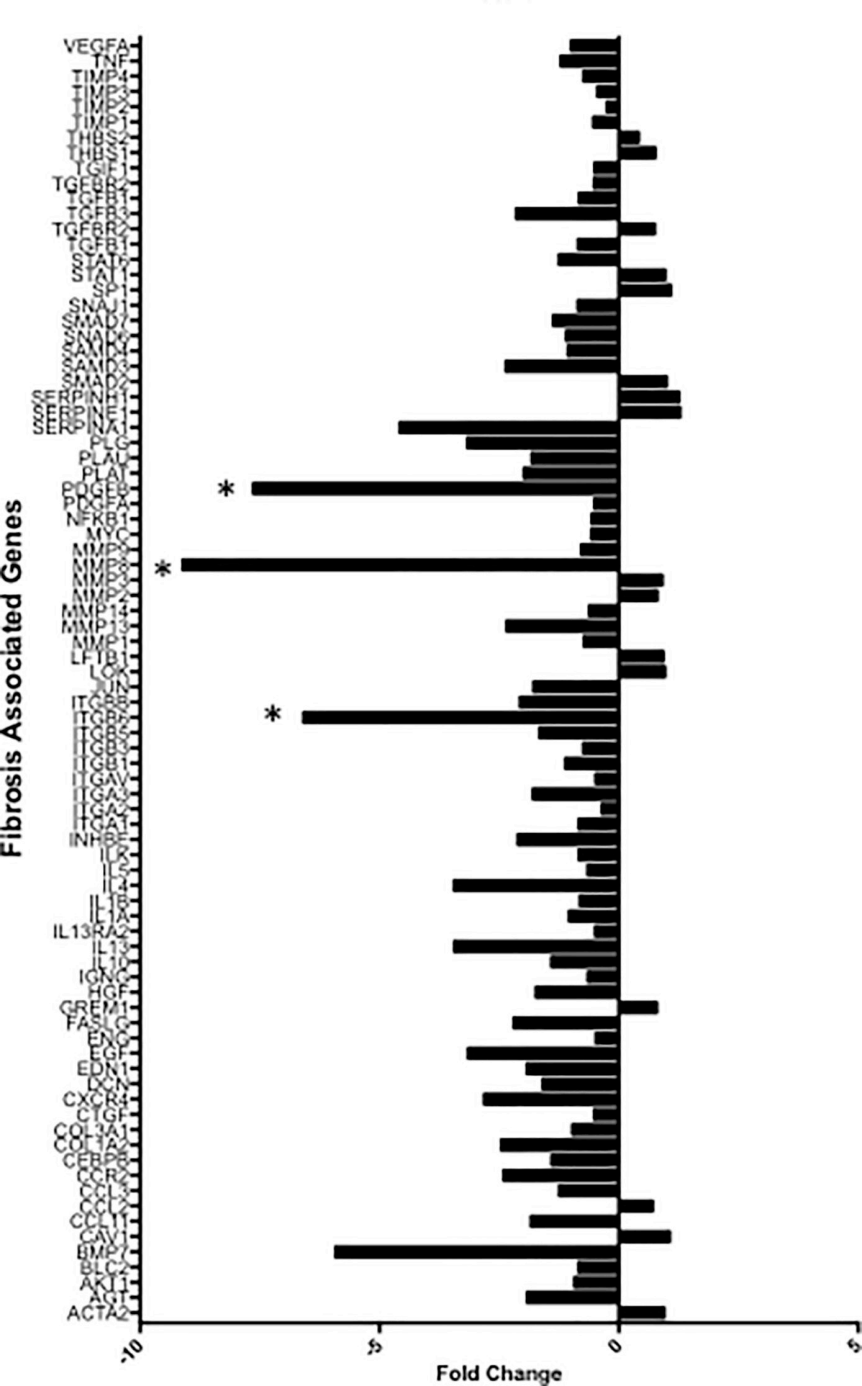
Bar Chart showing fold change difference in fibrosis related gene expression to house keeping genes of adipose derived stem cell scleroderma fibroblast co-culture (ADSC-SSc Co Culture) versus scleroderma fibroblast monoculture (SSc mono). [A] The majority of the fibrosis related genes were downregulated. The greatest fold change in expression was seen in genes marked with *including Matrix metalloproteinase-8 (MMMP-8), Platelet derived growth factor-β (PDGF-β) and Integrin Subunit Beta 6 (ITG-β6). Average fold change of co-culture versus monoculture of three independent experiments. Negative values = Decreased fold change in expression; Positive values = Increased fold change in expression.

Of the 84 fibrosis genes 68 fibrosis pathway specific genes was significantly down regulated in ADSC-SSc co-culture compared to SSc monoculture (Fig 5, S1 Fig) (p < 0.001). The greatest difference in fold change of gene expression compared to house keeping genes was seen in (1) Matrix metalloproteinase-8 (MMMP-8) gene expression, with a 9.18 fold reduction (2) Platelet derived growth factor-B (PDGF-B) gene expression, with a 7.63 fold reduction and (3) Integrin Subunit Beta 6 (ITG-β6) gene expression with a 6.5 fold reduction (Fig 5).

## 4. Discussion

In this study, we demonstrated a significant clinical improvement in orofacial fibrosis in SSc, previously regarded as a disease manifestation without effective therapy. Fibrosis is a cardinal feature of SSc that is often regarded as a prototypic fibrotic disease. In early stage disease there is marked fibrosis and thickening of the skin but as the disease progresses the skin may thin and become atrophic [38]. These changes in the skin are especially marked in the face and greatly affect facial appearance and function. These combined changes affect psychological wellbeing and have a major impact on quality of life and are regarded as more impacting than internal organ disease involvement by affected patients [13]. Clinical management is routinely based on self-administered home-based exercises that may temporarily improve mouth opening but have no long-term effect on function or quality of life [39]. Autologous stem cell enriched lipotransfer appears to be a successful intervention that improves the effects of facial fibrosis. It is a well-established and safe surgical technique. In our series out of 62 patients, we experienced only one case of infection in the recipient site (1.61%). This figure was similar to previous reports, where in a recent review paper on safety after lipotransfer, where the observed infection rate was between 0 and 3.6% [40].

We have demonstrated that injection of autologous stem cell-enriched lipotransfer into the peri-oral and facial tissue significantly improves mouth function, facial volumetric appearance with improved psychological outcome. To date, study cohorts for facial SSc have been limited by small sample sizes, limited outcome measures and short follow up [41–43]. In our series, we demonstrate significant improvement in the largest cohort of patients in the literature to date, using multiple validated outcome measures, with the longest mean follow-up of 12 months. The response durability was 100% at 6 months follow-up; 94% between 7 and 12 months follow up; and 66% after one year follow up.’

We also found that multiple sequential interventions produces cumulative benefits in both mouth function and aesthetics. The median of the number of treatments received was 3 (range 1–10). In this study cohort, we found that patients with lcSSc required less treatments (median 2, range 1–7) compared to patient with dcSSc (median 4, range 1–7).

We found that the psychological health of the patients in this study was significantly improved (Table 3). In contrast to previously published reports, our study was not limited to treating only the perioral area but also the cheeks, chin, nasolabial folds, and nose. This approach allowed for a better aesthetic outcome in terms of volume and facial elasticity that may have contributed to the psychological improvement in these patients (Table 3). We cannot rule out the possibility that the change in outcome may also be impacted by the Hawthorne effect. However, when we sub-divided the data, we found that multiple lipotransfer treatments further improved VAS and DAS24 scores that are related to aesthetic concerns (S1 Table) implying benefit due to the intervention.

This study takes into account the heterogeneity of this disease. Clinical heterogeneity can make optimum treatment of SSc difficult as the response to treatment can so often be heterogenous. LcSSc and dcSSc subsets differ in the rate of disease progression, degree of inflammation and extent of skin fibrosis [44]. When we compared the outcome measures of lcSSc (n = 36) and dcSSc (n = 26) subsets in this patient series we found that there was no difference in the response of these clinically diverse subsets to the intervention.

Immunological activity in SSc is a key potential stimulus to fibrosis [38,44]. As a result, the majority of treatment approaches to SSc are immunosuppressive. When we compared the outcome measures we found that there was no difference in the response of cases receiving concurrent immunosuppression. Thus, although the postulated effector cells, the ADSCs, are immunomodulatory it appears that the possible anti-fibrotic effect of ADSC-rich lipotransfer is independent of immunosuppression in this patient cohort. Immunosuppression is also thought to impair wound healing and is inadvisable to ensure optimal surgical outcome. However, we found that it had no impact on surgical outcome in this patient group.

Disrupted tissue homeostasis caused by excessive matrix remodeling and reduced remodelling leads to a loss in connective tissue that causes the skin to become atrophic and retract [38]. This often leads to facial volume loss, pronounced vertical furrows and thinning and retraction of the lips. Autologous stem-cell enriched lipotransfer restored peri-oral volume and lip movement with improved aesthetics by reducing vertical furrows (Fig 1). There was a higher resorption rate of the injected lipoaspirate in the mobile peri-oral area (Fig 2). Despite this higher resorption rate, significant improvements in mouth function were maintained in this area over time (Table 2, Fig 1). Greater improvements in mouth function were recorded in patients that received ≥3 treatments compared to ≤2 treatments implying a cumulative anti-fibrotic effect (Table 2). A previous short report suggested that there was no correlation between fat volume injected and potential antifibrotic effect [43], indicating a trophic, paracrine or regenerative component of autologous lipotransfer.

While the effector cell or mechanism is unclear, adipose tissue-derived lipoaspirates contain a heterogenous population of cells including adipose derived stem cells (ADSCs) [45]. In a previous study, we successfully isolated, cultured and characterized ADSCs from SSc patients and found them to be functional and phenotypically similar to healthy matched ADSCs although the proliferation and migration of SSc ADSCs was found to be reduced in comparison to ADSCs from controls [28]. The immunomodulatory and angiogenic effects of ADSCs are well documented [25,28]. It has also been suggested that ADSCs have antifibrotic properties through secretion of antifibrotic factors, matrix metalloproteinases and by modulating certain pro-fibrotic factors [45–48].

The ADSCs in this study suppressed the viability and proliferation of the SSc-HDF in culture in direct and indirect culture but not when using conditioned medium (Fig 3). These findings may suggest that this effect is by a paracrine effect mediated by soluble factors released by ADSCs. Other studies have found that ADSCs suppress proliferation of fibroblasts due to paracrine signalling [49–52]. However, the exact mediators that cause this effect is unknown. The decrease in proliferation using CM may not have been observed as the responsible mediators secreted by the ADSCs may have too short half-life or present at too low concentrations [52].

The protein secretion of TGF-β1 and CTGF was significantly lower over 14 days in SSc co-culture compared to SSc monoculture in direct and indirect analysis (Fig 4). By day 14 direct co-culture demonstrated significantly higher protein secretion of CTGF and TGF-β1 by the SSC-ADSC co-culture compared to indirect co-culture (p < 0.01). The data suggests the influence of ADSCs may suppress fibrosis through paracrine signaling. Few studies have also shown that ADSC suppress TGF-β1 leading to the regression of fibrosis [49–50]. Sun *et al* demonstrated that ADSSC alleviate radiation induced muscular fibrosis through the suppression of TGF-β1 expression in a rabbit model [53]. Hitwatashi *et al* demonstrated that ADSCs were able to reverse the vocal fold scarring through the suppression of TGF-β1 signalling *in vitro* [54]. However, the exact mechanism by which ADSCs interfere with TGF-β1 expression is still unknown. Fewer studies have examined the effect of ADSC on CTGF secretion (Fig 4). Rivera-Valdes *et al* demonstrated that ADSCs could reverse chronic kidney fibrosis through the suppression of CTGF gene expression, in addition to IL6, IL10- TNF and TGF-β1 [55]. Similarly, liver fibrosis was reversed by ADSCs intravenous injection through the down regulation of pro-collagen alpha1, CTGF and α-SMA mRNA [56].

The qPCR analysis supported these findings demonstrating reduction in TGF-β1, TGF-β2, TGF-β3 and their receptors. The qPCR analysis also demonstrated reduction of all fibrosis associated genes, providing further evidence that the lipotransfer may provide an anti-fibrotic effect. Interestingly in this study, MMP-8 and PDGF-BB were also found to be significantly decreased. Various MMPs are over-expressed in scleroderma, which can promote ECM degradation and the release of TGF-β1 [57]. MMP-8 has been implicated in the pathogenesis of fibrosis [58]. The role of MMP-8 in scleroderma is currently unknown. However, MMP-8 has been shown to have anti-inflammatory and pro-fibrotic activities in lung fibrosis [58]. Graig et al found that MMP-8 promoted lung fibrosis by reducing lung levels of Ip-10 and Mip-1α [58]. Suppression of MMP-8 could be one effect by which the lipotransfer suppressed dermal fibrosis in this study. PDGF are mitogenic and chemo-attractants to myofibroblasts and promote TGF-β1 signalling [59]. Suppression of this growth factor could be anti-fibrotic through the inhibition of ECM deposition by altering the TGF-β1 signalling pathway [59]. In summary, lipotransfer may reduce dermal fibrosis through the suppression of fibroblast proliferation and down regulation of collagen synthesis by altering the protein and gene expression key regulators of the fibrosis pathway including TGF-β1, MMP-8 and PDGF-ββ. Future work is needed to understand the how the TGF-β1 signalling pathway is modulated by ADSCs using knock out gene assays and protein analysis.

There are certain limitations to this study. This study was performed without a control group, hence we cannot rule out a placebo effect. To overcome this a prospective randomized controlled clinical trial will be performed to validate these findings. However, this study has provided an understanding into the progression of the scleroderma disease following lipotransfer, insight into appropriate methodology and measurement tools. This study has also provided a foundation by which a randomized control trial can be performed.

This study has evaluated the effect of the ADSC cell within the adipose tissue on SSc- fibroblasts to understand the mechanism by which it reverses fibrosis. Whilst the *in vitro* data has provided significant evidence that the effect of lipotransfer may be mediated by the ADSC effector cell within it, there are multiple other cell types within the lipoaspirate that may be contributing to the effect. Hence, future work will evaluate all cell types within the lipoaspirate to gain further insight into the mechanism by which lipotransfer may reverse fibrosis.

The SSc and ADSCs in this study were isolated from different donor participants. As ADSCs have shown to have some immunodulatory effect [60], future work will use ADSC and SSCc fibroblast from matched donors with a larger cohort to more closely mimic the clinical scenario.

## 5. Conclusions

This study reports an innovative and effective intervention that improves the effects of oro-facial fibrosis in SSc. Due to its complex pathogenesis and heterogeneity, the successful translation of therapies for SSc is a challenge. Successful treatment of SSc is likely to require targeting of multiple biological pathways and mediators [61–64]. Autologous stem cell enriched lipotransfer offers a potentially effective regenerative option to treat oro-facial fibrosis in SSc that operates independently of immunosuppression and disease subset.

## Supporting information

S1 Table Effect of multiple lipotransfer treatments on psychological outcomes Data are presented as mean ±SD, p≤0.05 was considered significant. Psychological outcomes were evaluated by self-report questionnaires. *VAS* Visual Analog Scale, *DAS24* Derriford Appearance Scale, *BFNES* Brief Fear of Negative Evaluation Scale, *HADS-A* Hospital Anxiety and Depression Scale-Anxiety, *HADS-D* Hospital Anxiety and Depression Scale-Depression.
(DOCX)

S2 Table Grading of 2D photographs pre- and post-treatment with autologous stem cell enriched lipotransfer. (DOCX)

S1 Fig **[A] Bar Chart showing Fold Change in fibrosis related gene expression to house keeping genes of adipose derived stem cell scleroderma fibroblast co-culture (ADSC-SSc Co Culture) and scleroderma fibroblast monoculture (SSc mono).** The majority of fibrosis related genes for the ADSC-SSc co culture were downregulated compared to the SSc monolayers. Negative values = Decreased gene expression; Positive values = Increased expression. [B] **Table showing p values of difference in fold change in fibrosis related gene expression to house keeping genes of adipose derived stem cell scleroderma fibroblast co-culture and scleroderma fibroblast monoculture.** The difference in fold expression between co-culture and monoculture was highly significant for many of the fibrosis associated genes evaluated.
(TIFF)

## Abbreviations

SSc: Systemic Sclerosis
MHISS: Mouth Handicap in Systemic Sclerosis scale
VAS: Visual Analog Scale
DAS24: Derriford Appearance Scale
BFNES: Brief Fear of Negative Evaluation Scale
HADS-A: Hospital Anxiety and Depression Scale-Anxiety
HADS-D: Hospital Anxiety and Depression Scale-Depression.
ADSCs: Adipose-derived stem cells
RMS: root mean square
lcSSc: limited cutaneous systemic sclerosis
dcSSc: diffuse cutaneous systemic sclerosis (dcSSc)
