# Supplementary material for: Stem cell enriched lipotransfer reverses the effects of fibrosis in systemic sclerosis
Source: PLoS One. 2019 Jul 17;14(7):e0218068. doi: 10.1371/journal.pone.0218068 (PMC6636710; doi:10.1371/journal.pone.0218068)
Supplement: S1 Table — Psychological outcomes were evaluated by self-report questionnaires. VAS Visual Analog Scale, DAS24 Derriford Appearance Scale, BFNES Brief Fear of Negative Evaluation Scale, HADS-A Hospital Anxiety and Depression Scale-Anxiety, HADS-D Hospital Anxiety and Depression Scale-Depression. (DOCX) [file pone.0218068.s001.docx]

**Supplementary Table 1** **Effect of multiple lipotransfer treatments on psychological outcomes**

|  | **Change after ≤2 LT**  **Procedures (n=29)** | **Change after ≥3 LT**  **Procedures (n=33)** | **≤2 LT procedures vs**  **≥3 LT procedures** |
| --- | --- | --- | --- |
|  |  |  |  |
| VAS | 2.37 ± 3.0 | 4.6 ± 4.76 | p=0.0159 |
| DAS24 | 7.44 ± 6.83 | 16.15 ± 9.7 | p<0.0001 |
| BFNE | 3.31 ± 5.93 | 2.57 ± 2.20 | p=0.3836 |
| HADS-A | 2.65 ± 3.33 | 3.0 ± 3.30 | p=0.4328 |
| HADS=D | 2.06 ± 2.13 | 2.00 ± 3.92 | p=0.4862 |

Data are presented as mean ±SD, p≤0.05 was considered significant.

Psychological outcomes were evaluated by self-report questionnaires. *VAS* Visual Analog Scale, *DAS24* Derriford Appearance Scale, *BFNES* Brief Fear of Negative Evaluation Scale, *HADS-A* Hospital Anxiety and Depression Scale-Anxiety, *HADS-D* Hospital Anxiety and Depression Scale-Depression.
