# Supplementary material for: Stem cell enriched lipotransfer reverses the effects of fibrosis in systemic sclerosis
Source: PLoS One. 2019 Jul 17;14(7):e0218068. doi: 10.1371/journal.pone.0218068 (PMC6636710; doi:10.1371/journal.pone.0218068)
Supplement: S2 Table — (DOCX) [file pone.0218068.s002.docx]

**Supplementary Table 2** Grading of 2D photographs pre- and post-treatment with autologous stem cell enriched lipotransfer

| **Grading** | **% patients Pre-op** | **% patients Post-op** |
| --- | --- | --- |
| Severe | 26 | 0 |
| Severe-Moderate | 10 | 17 |
| Moderate | 57 | 13 |
| Moderate-Mild | 7 | 30 |
| Mild | 0 | 40 |
