# Supplementary figures and images for: Stem cell enriched lipotransfer reverses the effects of fibrosis in systemic sclerosis
Source: PLoS One. 2019 Jul 17;14(7):e0218068. doi: 10.1371/journal.pone.0218068 (PMC6636710; doi:10.1371/journal.pone.0218068)

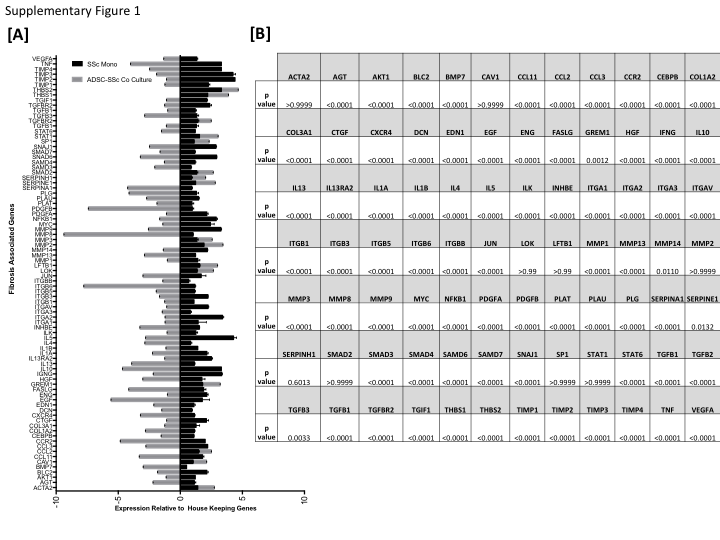

Supplement: S1 Fig — [A] Bar Chart showing Fold Change in fibrosis related gene expression to house keeping genes of adipose derived stem cell scleroderma fibroblast co-culture (ADSC-SSc Co Culture) and scleroderma fibroblast monoculture (SSc mono). The majority of fibrosis related genes for the ADSC-SSc co culture were downregulated compared to the SSc monolayers. Negative values = Decreased gene expression; Positive values = Increased expression. [B] Table showing p values of difference in fold change in fibrosis related gene expression to house keeping genes of adipose derived stem cell scleroderma fibroblast co-culture and scleroderma fibroblast monoculture. The difference in fold expression between co-culture and monoculture was highly significant for many of the fibrosis associated genes evaluated. (TIFF) [file pone.0218068.s003.tiff]
